# Functional Expression of TRPV4 Channels in Human Collecting Duct Cells: Implications for Secondary Hypertension in Diabetic Nephropathy

**DOI:** 10.1155/2012/936518

**Published:** 2012-09-20

**Authors:** Claire E. Hills, Rosemary Bland, Paul E. Squires

**Affiliations:** ^1^School of Life Sciences, The University of Warwick, Coventry CV4 7AL, UK; ^2^Warwick Medical School, The University of Warwick, Coventry CV4 7AL, UK

## Abstract

*Background*. The Vanilloid subfamily of transient receptor potential (TRPV) ion channels has been widely implicated in detecting osmotic and mechanical stress. In the current study, we examine the functional expression of TRPV4 channels in cell volume regulation in cells of the human collecting duct. *Methods*. Western blot analysis, siRNA knockdown, and microfluorimetry were used to assess the expression and function of TRPV4 in mediating Ca^2+^-dependent mechanical stimulation within a novel system of the human collecting duct (HCD). *Results*. Native and siRNA knockdown of TRPV4 protein expression was confirmed by western blot analysis. Touch was used as a cell-directed surrogate for osmotic stress. Mechanical stimulation of HCD cells evoked a transient increase in [Ca^2+^]_*i*_ that was dependent upon thapsigargin-sensitive store release and Ca^2+^ influx. At 48 hrs, high glucose and mannitol (25 mM) reduced TRPV4 expression by 54% and 24%, respectively. Similar treatment doubled SGK1 expression. Touch-evoked changes were negated following TRPV4 knockdown. *Conclusion*. Our data confirm expression of Ca^2+^-dependent TRPV4 channels in HCD cells and suggest that a loss of expression in response to high glucose attenuates the ability of the collecting duct to exhibit regulatory volume decreases, an effect that may contribute to the pathology of fluid and electrolyte imbalance as observed in diabetic nephropathy.

## 1. Introduction

Diabetic nephropathy (DN) is the leading cause of end-stage renal disease and subsequent entry into renal replacement programmes. Characterized by structural and functional disturbances, patients with DN can exhibit renal hypertrophy, fibrosis, altered glomerular filtration rate, glomerular hypertension, proteinuria, and systemic hypertension [1]. Maintaining cellular integrity is critical for normal cell function, but renal epithelial cells are exposed to constant fluctuations in filtrate flow and osmolality. Tubular cells have developed a number of mechanisms to compensate for cell volume changes induced by alterations in filtration rates and osmolarity. Two such complementary mechanisms are the serum and glucocorticoid-inducible kinase (SGK) and the mechanosensitive transient receptor potential (TRP) channel. Activated by cell shrinkage, SGK1 is one of the key regulators of Na^+^ reabsorption and is responsible for the insertion of the epithelial sodium channel (ENaC) into the apical membrane of the collecting duct (reviewed in [2]). In models of diabetic nephropathy, SGK expression is exacerbated [3, 4]. It has been suggested that accentuated increase in SGK-driven Na^+^ reabsorption contributes to the subsequent development of secondary hypertension observed in some patients with diabetic nephropathy.

The Vanilloid subfamily of TRP (TRPV) ion channels is implicated in detecting and transducing responses to a broad range of stimuli that include mechanical stress, heat, and pain (reviewed in [5]). Although we have a good understanding of how glucose alters SGK expression and function, as a potential counter-regulatory mechanism of SGK in the kidney, there is only sparse data pertaining to the effects of glucose on the calcium permeable TRPV channels, known to be activated in response to increased hypotonicity.

Mammalian TRP channels have been organised into protein subfamilies based on sequence identity [6]. Within this subfamily of related proteins, the Ca^2+^ permeable TRPV4 channel is expressed abundantly in water impermeant segments of the nephron, including the cortical collecting duct [7], where it acts as a sensor for both osmolarity and shear stress [8]. The rise in cytosolic calcium ([Ca^2+^]_*i*_) that follows hypoosmotic-induced cell swelling has been demonstrated in a number of volume-regulating cell types, including, bronchial epithelial cells [9], chondrocytes [10], and bladder urothelium [11]. The hypotonic activation of TRPV4, with a concomitant rise in [Ca^2+^]_*i*_, has implicated this Ca^2+^ permeable cation channel as a possible mediator in cellular osmoreception in aiding cell volume recovery. Dysregulation of TRPV4 channel functional expression may have severe repercussions for epithelial integrity.

In the current study, we have used a novel model cell system of the human collecting duct (HCD-cells) to confirm expression and function of TRPV4 channels. We have examined the effect of high glucose on TRPV4 and SGK1 expression in an attempt to better elucidate the role of these regulatory proteins in maintaining cell volume under physical stress and thus preventing the development of secondary hypertension which is often observed in DN.

## 2. Materials and Methods

### 2.1. Cell Culture

HCD cells were derived from normal human kidney cortex and immortalized with SV-40 virus. Clones were selected using the monoclonal antibody, Ab272, which specifically recognizes collecting duct principal cells. HCD cells (passages 18–30) were maintained in DMEM/Hams F-12 medium (GIBCO, Invitrogen), supplemented with 2% fetal calf serum (FCS), glutamine (2 mmol/L), 15 mmol/L HEPES, transferrin (5 *μ*g/mL), Na_2_SeO_3_ (5 ng/mL), insulin (5 *μ*g/mL), and dexamethasone (5 × 10^–8^ M). Cells were grown at 37°C in a humidified atmosphere of 5% CO_2_ in air. Prior to treatment, cells were cultured in DMEM/Hams F-12 low glucose (5 mM) for 48 hours. Basal (5 mM) glucose and high glucose (25 mM) culture media was generated as described previously [12]. For calcium experiments, cells were seeded onto 3-Aminopropyltriethoxysilane-(APES-) (Sigma, Poole, UK) treated coverslips and used within 1 day of plating. In all experiments, cells were serum starved overnight before agonist stimulation. For immunocytochemistry and microfluorimetry experiments, cells were seeded onto 3-Aminopropyltriethoxy-silane (APES) (Sigma, Poole, UK) treated coverslips and used within 2 days of plating.

### 2.2. Analysis of mRNA Expression

RNA was prepared from 80% confluent HCD cells by acid-guanidinium extraction [13] using a genelute mammalian total RNA miniprep kit (Sigma) following the manufactures instructions. Complementary DNA was synthesized by reverse transcription using a Promega Reverse Transcription System following an adapted method. Briefly, 1 *μ*g of total RNA and 0.5 *μ*g of random hexamers, in a final volume of 11 *μ*L, were incubated at 70°C for 5 minutes, and then allowed to cool slowly to 25°C. Primer extension was then performed at 37°C for 60 minutes following the addition of 1x (final concentration) reaction buffer, containing 50 mmol/L Tris-HCl (pH 8.3), 50 mmol/L KCl, 10 mmol/L MgCl_2_, 10 mmol/L dithiothreitol and 0.5 mmol/L spermidine, 1 mmol/L (final concentration) of each dNTP, 40 U of rRNAsin ribonuclease inhibitor, and 15 U of AMV reverse transcriptase in a final volume of 20 *μ*L. The RT mixture was heated to 95°C for 5 minutes, then 4°C for 5 minutes. An aliquot of 4 *μ*L was used in subsequent polymerase chain reaction (PCR) reactions.

### 2.3. PCR Amplification of cDNAs

Amplification of specific cDNAs was carried out using the primers listed in Table 1. PCR reactions (20 *μ*L) were set up containing 1.5 mmol/L MgCl2, 0.2 mmol/L of each dNTP, 0.5 *μ*M of each primer, and 1 U of Taq DNA polymerase (Bioline). Amplification of samples was performed using an initial denaturation step of 95°C (5 minutes) followed by either 34 (TRPV4) or 35 (SGK1) cycles consisting of one minute of denaturing at 95°C, one minute of annealing at the required temperature (Table 1), and a one-minute extension at 72°C. A final elongation step of 72°C for seven minutes was included in all PCR amplifications.

### 2.4. Analysis of Protein Expression

The preparation of cytosolic proteins and their subsequent separation by gel electrophoresis and electroblotting onto Immobilon P membrane (Millipore, Watford, UK) were as described previously [14]. Briefly, proteins (5 *μ*g) were separated by sodium dodecyl sulphate (SDS) polyacrylamide gel electrophoresis (4.5% stacking gel, 7.5% or 10% resolving gel) at 200 volts for 50 minutes in electrophoresis buffer containing 25 mmol/L Tris, 192 mmol/L glycine, and 0.1% (wt/vol) SDS. Proteins were transferred onto Immobilin P membrane in transfer buffer (25 mmol/LTris, 192 mmol/L glycine, and 20% (vol/vol) methanol) for 1 hour at 100 volts; 4°C. Following protein transfer, membranes were blocked in PBS-T (PBS plus 0.1% Tween-20) containing 20% (wt/vol) nonfat milk powder (Marvel, Premier Brands, Stafford, UK) for 1 hour at 25°C and then washed with PBS-T for 15 minutes. Filters were analyzed with specific polyclonal antibodies against human TRPV4 (The Binding Site Ltd., Birmingham, UK) diluted in PBS-T (0.05%) at 1 : 1000 or SGK (The Binding Site Ltd., Birmingham, UK) diluted in PBS-T (0.05%) at 1 : 3000. After three 10-minute washes in PBS-T, the membranes were incubated with the secondary antibody horseradish peroxidase-conjugated antisheep (diluted 1 : 30,000) in PBS-T (0.05%) for 60 minutes at 25°C followed by three 10-minute washes in PBS-T. Specific proteins were detected using ECL detection reagent chemiluminescence system (Amersham Biosciences) and were visualized after exposure of membranes to X-ray film for 1–10 minutes. Control experiments were included where primary antibody was omitted, and filters were exposed to secondary antibody and ECL detection.

### 2.5. Knockdown of TRPV4 Expression

Cells were allowed to grow to 40% confluence in 6 well plates or APES-treated coverslips. Knock-down of TRPV4 expression was achieved by two different siRNA protocols.

(1) Transfection of cells with a TRPV4 specific siRNA (Invitrogen). Transfection of siRNAs was carried out using lipofectamine (Invitrogen) following manufacturers instructions. Briefly, lipofectamine and siRNAs were diluted into OptiMEM medium (Invitrogen). Diluted lipofectamine lipids were mixed with diluted siRNAs and the mixture was incubated for 30 minutes at room temperature for complex formation. Mixtures were further diluted in OptiMEM and added to each well such that the final concentration of siRNAs was 80 nM. Cells were harvested and assayed 24 hours after transfection. Negative controls included untransfected cells, lipid alone, and scrambled siRNA (Santa Cruz).

(2) The siLentGene U6 Cassette RNA Interference System (Promega). Specific TRPV4 and scrambled sequences were selected using the Promega design tool. U6 expression cassettes were constructed following the manufacturers instructions. Transfection of the U6 DNA expression cassettes was performed using siLentGene transfection reagent (Promega) such that the final concentration of cassettes was 160 ng/mL. In order to localise transfected cells, cells were cotransfected with RFP (pDsRed2-C1). Cells were harvested and assayed 72 hours after transfection. Negative controls included untransfected cells, lipid alone, RFP alone, and a scrambled siRNA. In each case, TRPV4 knockdown was confirmed by Western blot analysis as described above.

### 2.6. Single-Cell Microfluorimetry

HCD cells seeded and grown overnight on APES-coated coverslips were loaded for 30 minutes at 37°C with 2.5 *μ*M of the Ca^2+^ fluorophore Fura-2/AM (Sigma, UK). Coverslips were washed and placed in a steel chamber, the volume of which was approximately 500 *μ*L. A single 22 mm coverslip formed the base of the chamber, which was mounted into a heating platform on the stage of an Axiovert 200 Research Inverted microscope (Carl Zeiss Ltd., Welwyn Garden City, UK). All experiments were carried out at 37°C using unsupplemented DMEM/Hams F-12 as the standard extracellular medium. Cells were illuminated alternatively at 340 nm and 380 nm using a Metafluor imaging workbench (Universal Imaging Corp Ltd., Marlow, Bucks, UK). Emitted light was filtered using a 510 nm long-pass barrier filter and detected using a Cool Snap HQ CCD camera (Roper Scientific). Changes in the emission intensity of Fura-2 expressed as a ratio of dual excitation were used as an indicator of changes in [Ca^2+^]_*i*_ using established procedures. For Ca^2+^ free experiments cells were bathed in calcium-free media supplemented with 1 mM EGTA. To assess the contribution made by intercellular calcium stores HCD cells were bathed in Ca^2+^-free media following a 60 minute preincubation with thapsigargin (1 *μ*M). Cells were then subject to touch evoked stimulation and [Ca^2+^]_*i*_ levels recorded. Data was collected at 3-second intervals for multiple regions of interest in any one field of view. All records have been corrected for background fluorescence (determined from cell-free coverslip).

### 2.7. Mechanical Stimulation of HCD Cells

Individual cells within a cluster (6–12 cells/cluster) of Fura-2-loaded cells were stimulated via touch using a femtotip electrode delivery system (Eppendorf, Hamburg, Germany). Maintained Fura-2 fluorescence confirmed integrity of the cell membrane.

### 2.8. Analysis

Autoradiographs were quantified by densitometry using TotalLab 2003 (NonLinear Dynamics, Durham, NC, USA). Where data was quantified, the nonstimulated, low-glucose control condition was normalized to 100% and data from all other experimental conditions compared to this. Statistical analysis of data was performed using a one-way ANOVA test with a Tukey's multiple-comparison posttest. Data are expressed as mean ± SEM, and “*n*” denotes the number of experiments. Probability (*P*) < 0.05 was taken to signify statistical significance.

## 3. Results

### 3.1. Expression of TRPV4 and SGK1 in HCD Cells

**Figure 1:**
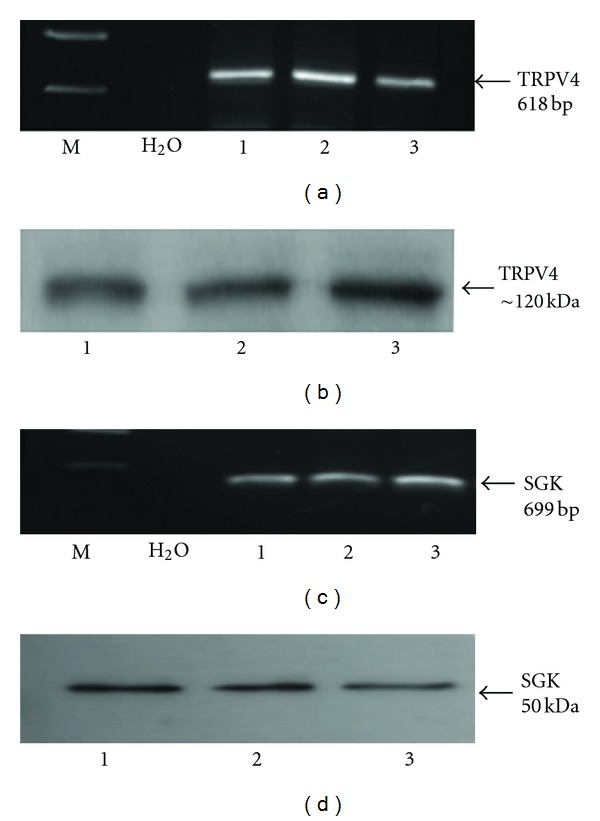
Expression of TRPV4 mRNA and protein in HCD-cells. (a) and (c) represent RT-PCR analysis using primers specific for human TRPV4 and SGK, respectively. PCR products of 618 bp and 699 bp were observed in three RNA preparations (1, 2, and 3) corresponding to mRNA expression for TRPV4 and SGK in HCD cells. Negative controls included both water and samples in which AMV enzyme had been omitted from the reverse transcription reaction (data not shown). Western blot analyses of HCD cell lysates ((b) and (d) 5 *μ*g protein/lane) using an antibody against human TRPV4 and SGK confirmed the presence of the protein. A protein band of approximately 120 KDa (TRPV4) and 50 KDa was detected. Controls included TRPV4 and SGK antibody preabsorbed with a 100-fold excess of immunizing peptide (data not shown).

Studies confirmed the presence of both TRPV4 and SGK1 mRNA and protein expression in HCD cells. RT-PCR analysis of three RNA preparations from HCD cells revealed PCR products representative of TRPV4 (Figure 1(a)) and SGK1 (Figure 1(c)) mRNAs, respectively. To confirm that all sets of mRNA were appropriately translated, protein expression was determined by Western blotting (Figures 1(b) and 1(d)). Western blot analyses revealed bands at approximately 120 kDa and 50 kDa, representatives of those expected for TRPV4 and SGK, respectively.

### 3.2. Touch-Evoked Changes in [Ca^2+^]_*i*_ in HCD Cells

**Figure 2:**
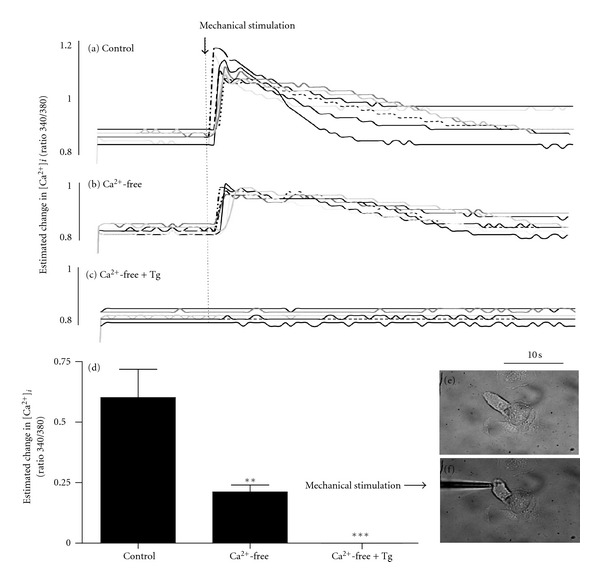
Changes in [Ca^2+^]_*i*_ in HCD-cells evoked by mechanical stimulation. In the presence of extracellular calcium (a control), mechanical stimulation of an individual cell, within a cluster, elicits an increase in [Ca^2+^]_*i*_. The rise in [Ca^2+^]_*i*_ rapidly propagates into coupled cells. Removal of extracellular calcium (b) reduces the basal-to-peak change in mechanically evoked [Ca^2+^]_*i*_, but does not negate the response. Depletion of Ca^2+^ stores via preincubation of the Ca^2+^-ATPase inhibitor, thapsigargin (Tg 1 *μ*M; 30 min, (c) in the absence of extracellular calcium, completely prevents mechanically evoked changes in [Ca^2+^]_*i*_. (d) shows mean basal-to-peak data from several experiments (*n* = 4) and indicates a role for both intra- and extracellular calcium in mediating mechanically evoked Ca^2+^-signalling in HCD-cells. Data is represented as an estimated change in [Ca^2+^]_*i*_ recorded as a ratio of 340/380 nm excitation for fura-2. Statistical analysis ((d); *P* < 0.01 control versus Ca^2+^-free and *P* < 0.001 control versus Ca^2+^-free + Tg).

Physical stimulation of a single HCD cell evoked an increase in cytosolic calcium (Figure 2(a)). The response was rapid in onset but transient, with [Ca^2+^]_*i*_ returning to basal levels within 60 ± 10 sec following the initial stimulation and without removal of the stimulating electrode. The rapid transmission of a [Ca^2+^]_*i*_ signal away from the point of stimulation illustrates cooperativity between HCD-cells and is indicative of the high degree of cell-to-cell communication previously demonstrated [12] for these cells (data representative of 5 separate experiments). To examine the role of Ca^2+^ influx in mediating touch evoked changes in [Ca^2+^]_*i*_, cells were bathed in calcium-free media (+EGTA; 1 mmol/L). One touch stimulation of a single cell within a cell cluster still generates an increase in [Ca^2+^]_*i*_ that propagated into neighbouring cells (Figure 2(b)). However, the basal-to-peak amplitude of this response (0.21 ± 0.03%) was only 35% of that obtained in the presence of extracellular calcium (0.60 ± 0.121%; *P* < 0.01   *n* = 6 separate experiments; see Figures 2(b) and 2(d)). Preincubation of cell clusters in Ca^2+^-free media containing the Ca^2+^-ATPase inhibitor thapsigargin (Tg 1 *μ*M) depleted intracellular stores [15] and completely abolished touch-evoked changes in [Ca^2+^]_*i*_ as expected (Figures 2(c) and 2(d)).

### 3.3. Glucose-Induced Downregulation in TRPV4 Expression Is Paralleled by an Upregulation in SGK

**Figure 3:**
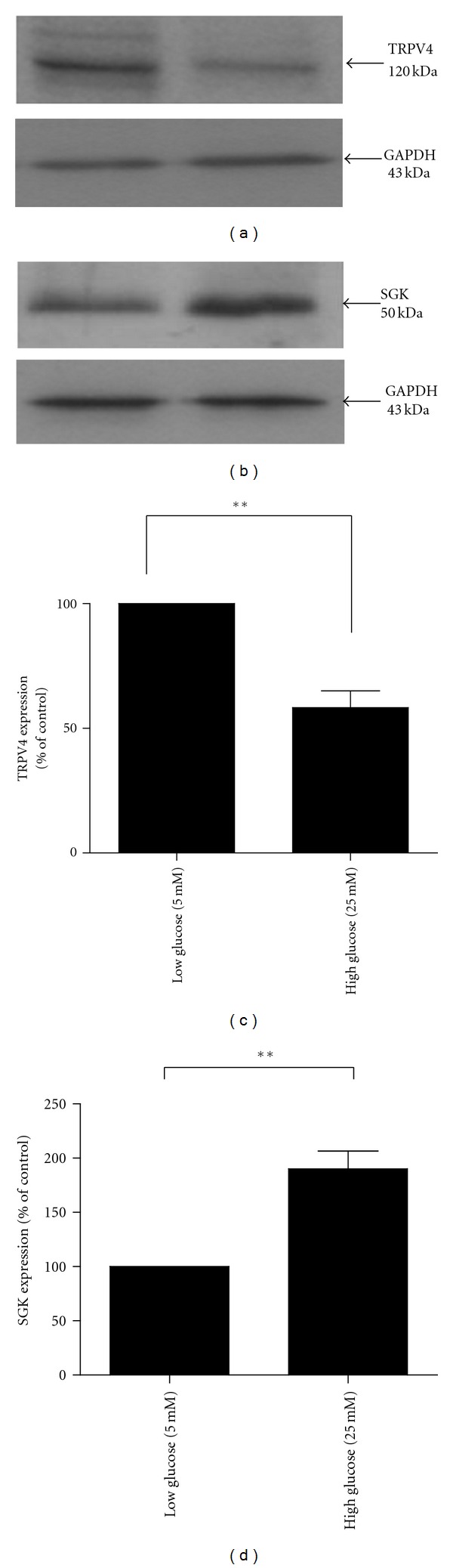
The effect of glucose on TRPV4 and SGK1 protein expressions in HCD cells. HCD-cells were incubated in 5 mM and 25 mM glucose for 48 hours. (a) Representative Western blot analysis using an anti-TRPV4 antibody; (c) analysis of changes in TRPV4 protein expression. Results represent mean ± SEM; *n* = 4; **P < 0.01. (b) Representative Western blot analysis using an anti-SGK antibody; (d) analysis of changes in SGK protein expression. Results represent mean ± SEM; *n* = 4; **P < 0.01.

To examine the effect of elevated glucose on TRPV4 and SGK expression, HCD cells were incubated in high glucose (25 mM) for 48 hours and expression levels of TRPV4 and SGK determined by western blotting. HCD cells grown under high-glucose conditions exhibited a 54% reduction in TRPV4 expression to 46%  ± 6.6% as compared to control (5 mM) at 48 hrs (*n* = 3, *P* < 0.01, see Figures 3(a) and 3(c)). Contrary to the effect on TRPV4, high glucose evoked a 90%  ± 16.5% increase in SGK expression as compared to control at 48 hours, respectively (Figures 3(b) and 3(d)) (*n* = 3, *P* < 0.01). Mannitol (25 mM) was used as a control for the osmotic effects of high glucose and decreased TRPV4 expression at 48 hr by approximately 24%  ± 1.4% of the glucose-evoked change seen under identical experimental conditions (*P *= NS, data not shown).

### 3.4. Touch-Evoked Changes in [Ca^2+^]_*i*_ Are Mediated by TRPV4 Channels

**Figure 4:**
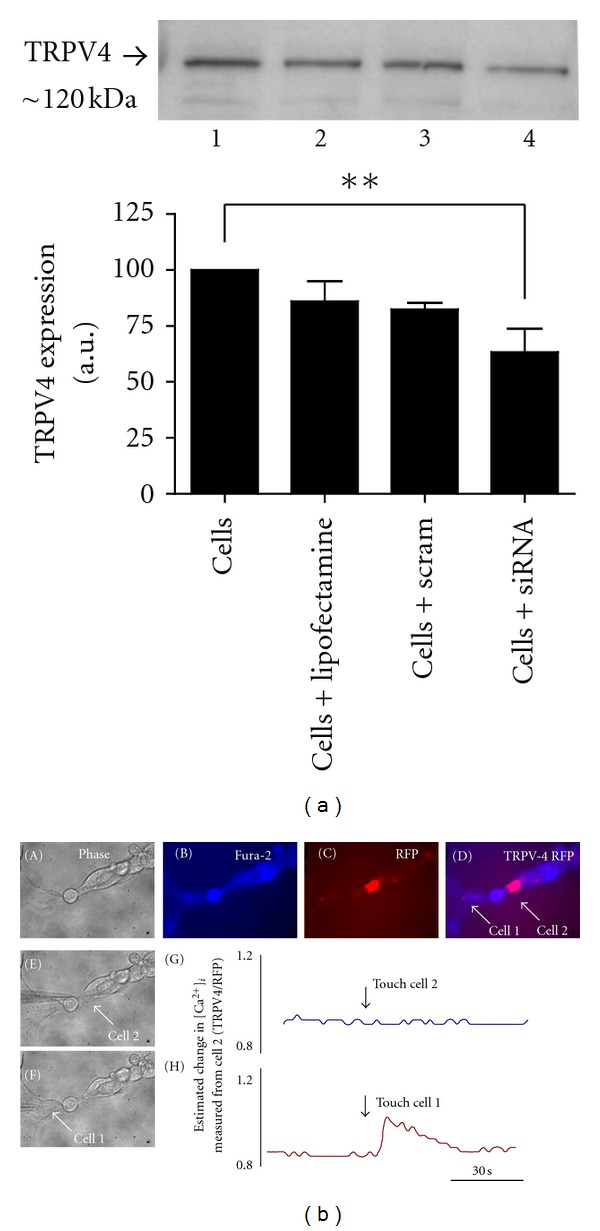
TRPV4 mediates mechanically evoked changes in [Ca^2+^]_*i*_. (a): western blot analyses of HCD cell lysates (5 *μ*g protein/lane) using an antibody against human TRPV4 (120 kDa) confirmed siRNA knock-down of TRPV4 expression in lane 4, as compared to control cells (lane 1, untransfected cells; lane 2, cells transfected with lipid alone; lane 3, cells transfected with scrambled siRNA). Knock-down reduced TRPV4 expression to approximately 60% of control. Results represent mean ± SEM; *n* = 3; **P < 0.01. In (b), a single cell within a cluster of HCD-cells can be identified following co-transfection with anti-TRPV4 siRNA and Red Fluorescent Protein (RFP) using the siLentGene U6 Cassette RNA Interference System (Promega; (b) C). The same cell cluster is visualised as an overlay image following loading with fura-2/AM ((b) D). In (b) F, a nontransfected cell (cell 1) is stimulated mechanically to evoke a rise in [Ca^2+^]_*i*_ ((b) H). The RFP-identified cell 2 ((b) D) fails to evoke a touch-evoked Ca^2+^-signal ((b) G).

Transiently transfecting cells with siRNA for TRPV4 significantly reduced TRPV4 protein expression in HCD-cells to approximately 60% of control as confirmed by western blot analysis (Figure 4(a) lane 4; representative of 4 separate experiments). Lipofectamine alone or scrambled siRNA did not reduce TRPV4 expression. Although encouraging, the level of downregulation was insufficient to assess functional responses within the population as a whole. To overcome this issue, an alternative strategy using a siLentGene Interference system (Promega) allowed cotransfection with Red Fluorescent Protein and anti-TRPV4, to identify single-transfected cells within cell clusters (Figure 4(b); representative of 4 separate experiments). Mechanical stimulation of a nontransfected cell, “cell-1”, (panel F) elicited a rapid increase in [Ca^2+^]_*i*_ (panel H). However, stimulation of an anti-TRPV4 cell (RFP-tagged “cell-2”; panel C, D and E) failed to evoke a change in [Ca^2+^]_*i*_ (panel G) as previously observed under control conditions. Transfection with lipid, RFP-alone or -scrambled siRNA sequences did not alter responses to touch (data not shown). These data suggest that knock-down of TRPV4 expression inhibits the ability of HCD-cells to detect deformation at the cell membrane in response to either osmotic or mechanical stress.

## 4. Discussion

Renal epithelial is constantly exposed to fluctuations in filtrate flow and osmolality. Any changes in cell volume as a result of these physical stresses must be minimized by appropriate counter-regulatory mechanisms. Dysregulated cell volume control will lead to altered cell volume, altered function, and ultimately tissue damage. During the progression of renal disease the loss of cells in the nephron is paralleled by compensatory hypertrophy of the remaining cells, an effect mediated by a change in sodium transport and regulated by an increase in cell volume [16]. These changes can instigate a number of downstream effector mechanisms that can influence epithelial integrity.

In the current study, touch was used as a cell-directed surrogate for osmotic stress in an attempt to confirm and further delineate a role for TRPV4 in stimulus-response coupling of physical stimuli in the collecting duct. Furthermore, we have investigated a role for glucose in the regulation of TRPV4 expression and discussed the implications that these findings may have on the ability of the collecting duct to detect and respond to osmotically induced signals under pathophysiological conditions such as diabetes. In our model system, we confirm expression of TRPV4 mRNA and protein and demonstrate that these human-derived cells detect and transduce touch through a transient rise in cytosolic calcium [Ca^2+^]_*i*_, a finding consistent with previous data linking membrane stretch in A6 cells to elevated [Ca^2+^]_*i*_, a key response in cell volume regulation [17]. The changes in [Ca^2+^]_*i*_ were mediated by both Ca^2+^-influx and –store release, and the response was negated by knockingdown the expression of TRPV4. The response of cells to hypoosmotic challenge is believed to be a two-step process involving detection of increased cell volume via increased membrane stretch, accompanied by alterations in intracellular solute content. The end goal is restoration of cell volume via a regulatory volume decrease (RVD), a Ca^2+^-dependent process [18–20]. The importance of Ca^2+^ is highlighted by the observation that cells of the thick ascending limb of Henle's loop (TALH) and human cervical cancer cells lose their ability to regulate cell volume in Ca^2+^-free media [21]. The origin of osmotically evoked changes in [Ca^2+^]_*i*_ in RVD varies between cell types and can depend on either Ca^2+^ influx alone [8] or have dual dependency on Ca^2+^ mobilisation from cytosolic stores [22], where store depletion has been shown to subsequently activate Ca^2+^ influx via store operated entry [20, 21]. Evidence from the current study supports a role for TRPV4 in regulating cell volume through both Ca^2+^ mobilisation and influx pathways. In the absence of extracellular calcium the basal-to-peak amplitude of touch-evoked changes in [Ca^2+^]_*i*_ was significantly lower than that evoked by the same stimulus in calcium containing conditions. Previous studies have suggested that the rise of [Ca^2+^]_*i*_ during cell swelling is a consequence of both TRPV4-mediated Ca^2+^ influx [23, 24], and Ca^2+^-induced Ca^2+^ release (CICR) from thapsigargin-sensitive intracellular stores [17]. Therefore, although Ca^2+^-influx is not essential in elicitating a rise in [Ca^2+^]_*i*_, Ca^2+^ entry accentuates the amplitude of the response and may be crucial in initiation of RVD [17, 25].

**Figure 5:**
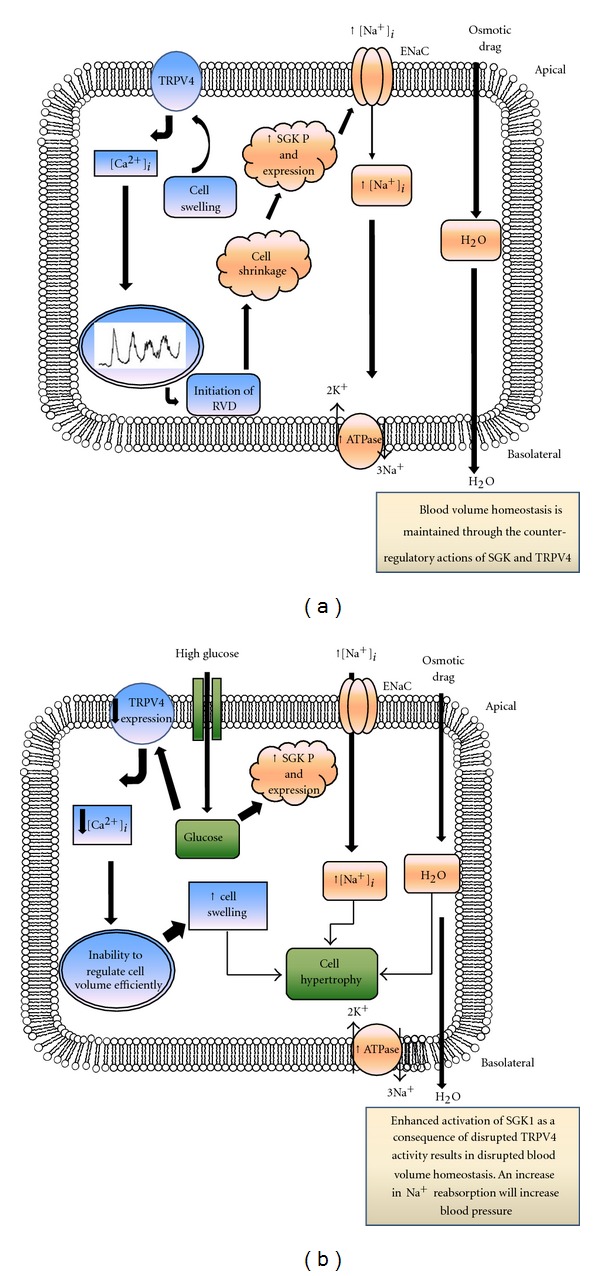
TRPV4 and SGK1 in the kidney. In healthy nondiabetic individuals, SGK1 is responsible for insertion and retention of ENaCs into the apical cell membrane, promoting ENaC-mediated Na^+^ reabsorption from the lumen of the cortical collecting duct (a). SGK1 also stimulates the Na^+^,K^+^-ATPase on the basolateral membrane. TRPV4 receptors will be activated as a counterregulatory mechanism, initiating a calcium-dependent response, which ensures activation of a regulatory volumes decrease. Through the actions of SGK1 and TRPV4 cell volume homeostasis is maintained. In a patient with diabetes, however, these counter-regulatory mechanisms may become compromised as a consequence of glucose-evoked changes in expression (b). SGK expression is increased in response to high glucose. Similarly, glycosuria may initiate cell shrinkage as water is lost to the lumen. Cell shrinkage is a key trigger for SGK1 activation. This will result in enhanced SGK1 activity, increased [Na^+^]_*i*_, and enhanced osmotic drag, all of which induce cell swelling. Whilst under healthy conditions this will be compensated for by a TRPV4-mediated regulatory cell volume decrease; in high glucose, a loss in the expression of this channel (b) will reduce sensitivity of the cell to deformation at the membrane and [Na^+^]_*i*_ will rise.

In diabetes, increases in circulating glucose often spill over into the urine. The resultant osmotic drag increases tubular flow within the nephron and increases the formation of a hyperosmotic urine. These physical changes induce cell shrinkage of renal epithelial cells and ultimately trigger activation of those compensatory mechanisms aimed at restoring cell volume. Activated in response to cell shrinkage, SGK1 is one of the key regulators of Na^+^ reabsorption in the nephron through regulation of the epithelial sodium channel (ENaC) [14]. Demonstrating elevated levels of expression in models of diabetic nephropathy with concomitant increased [Na^+^]_*i*_ levels, SGK has been proposed as a potential key mediator of increased Na^+^ reabsorption and the subsequent development of secondary hypertension observed in patients with diabetic nephropathy [3, 4]. Under nondiabetic healthy conditions, SGK1 acts to increase ENaC-mediated Na+ and water reabsorption, instigating a regulatory volume increase [26] (Figure 5(a)). However, in diabetes, and as confirmed by the current study, where hyperglycaemia dramatically increases SGK1 expression, without the intervention of rapid counter-regulatory mechanisms to alleviate exacerbated SGK-mediated Na^+^ retention, the individual would be prone to exaggerated sodium retention and secondary hypertension. TRPV4-mediated increases in [Ca^2+^]_*i*_ have been shown to initiate a cell regulatory volume decrease and thus counterbalance the hyperosmotic-inducted effects of SGK. However, in the current study we show that high glucose dramatically downregulates TRPV4 expression. Loss in TRPV4 will compromise the necessary counter-regulatory volume decrease essential to the cell if able to respond to a state of cell swelling induced by glucose-evoked increases in SGK1 expression (Figure 5(b)). In the present study, siRNA knockdown of TRPV4 expression negated touch-evoked changes in [Ca^2+^]_*i*_ further confirming the pivotal role for TRPV4 channels in detection and transduction of osmotic-like signals and demonstrating the potential consequences of a loss in expression over cell integrity and function under pathophysiological conditions such as diabetes.

In conclusion, the current study suggests that touch-evoked stimulation of HCD cells initiates a rise in [Ca^2+^]_*i*_ through TRPV4 channel stimulus-response coupling to thapsigargin-sensitive intracellular store release and Ca^2+^ entry; changes previously shown to be key mediators in instigation of cell volume regulatory mechanisms. As already discussed, cell volume regulation is critical to the overall integrity of both the cell and nephron. Inability therefore to respond appropriately to osmotic stimuli could have detrimental consequences for fluid and electrolyte balance in the kidney and may ultimately contribute to various renal pathologies, including diabetic nephropathy and end-stage renal disease.

## Figures and Tables

**Table 1:** Polymerase chain reaction (PCR) primers used to amplify TRPV4 mRNA. F: forward primer, R: reverse primer.

mRNA	Primers (5′→3′)	Direction	Annealing temp	Product size
TRPV4	CCCGTGAGAACACCAAGTTT TCACTCCAGGGCATTTCTTC	F R	58°C	618 bp
